# Physical exercise stimulates salivary secretion of cystatins

**DOI:** 10.1371/journal.pone.0224147

**Published:** 2019-10-24

**Authors:** Marcelo de Lima Sant’Anna, Leandro Teixeira Oliveira, Diego Viana Gomes, Sergio Tadeu Farinha Marques, D. William Provance, Martha Meriwether Sorenson, Verônica Pinto Salerno

**Affiliations:** 1 Institute of Medical Biochemistry, Federal University of Rio de Janeiro, Rio de Janeiro, Brazil; 2 Department of Physical Activity Biosciences, Federal University of Rio de Janeiro, Rio de Janeiro, Brazil; 3 Almirante Sylvio de Carmargo Training Center, Brazilian Navy, Rio de Janeiro, Brazil; 4 Naval Sports Commisson, Brazilian Navy, Rio de Janeiro, Brazil; 5 Center for Technological Development in Health, Oswaldo Cruz Insitute, Rio de Janeiro, Brazil; University of Pisa, ITALY

## Abstract

Physical exercise is known to activate the sympathetic nervous system, which influences the production of saliva from salivary glands. Our examination of saliva collected from highly trained athletes before and after a number of physical competititions showed an increase in the secretion of S-type cystatins and cystatin C as a subacute response to aerobic and anaerobic exercise. The elevation in salivary cystatins was transient and the recovery time course differed from that of amylase and other salivary proteins. An *in vitro* assay was developed based on a cell line from a human submandibular gland (HSG) that differentiated into acinus-like structures. Treatments with the β-adrenergic agonist isoproterenol caused a shift in the intracellular distribution of S-type cystatins and cystatin C, promoting their accumulation at the outer regions of the acinus prior to release and suggesting the activation of a directional transport involving co-migration of both molecules. In another treatment using non-differentiated HSG cells, it was evident that both expression and secretion of cystatin C increased upon addition of the β-adrenergic agonist, and these effects were essentially eliminated by the antagonist propranolol. The HSG cell line appears to have potential as a model for exploring the mechanism of cystatin secretion, particularly the S-type cystatins that originate primarily in the submandibular glands.

## Introduction

Saliva is a complex fluid containing proteins, carbohydrates, inorganic molecules, lipids, nucleic acids and water [1]. It can also include gingival exudate, cellular debris and a sampling of the microorganisms present in the mouth [2,3]. In humans, the larger glands (parotid, submandibular and sublingual) secrete up to 85% of the saliva [3,4,5], and the protein levels are regulated by physiological stimuli [3,6]. α-Amylase is a major component, and has long been a popular target for studying glandular secretion [7]; other proteins have received less attention.

The autonomic nervous system responds differentially to physical exercise [8]. While the major glands are innervated by sympathetic as well as parasympathetic nerves, which drive fluid secretion, sympathetic signaling predominates during exercise. This difference in signalling can lead to an increase in protein expression and secretion even as fluid secretion is reduced [3]. However, responses to stimuli differ from one gland to another, and the co-existence of different secretory pathways imposes additional variables [4]. Recent work has shown that the increased sympathetic stimulus induced by physical exercise can further modulate the roster of proteins secreted from salivary glands [9,10,11,12].

The multiple observations that exercise can alter the protein profile in saliva prompted us to explore the changes detectable in saliva from trained athletes. We readily identified proteins of the cystatin family, a group of cysteine proteases of ~16 kD, because they increased markedly with exercise, and are much smaller proteins than amylase. Previous studies have reported cystatins as a product primarily of submandibular glands, while amylase is derived mainly from parotid glands [13]. Intrigued by these differences, we conducted further analyses to determine the contributions to salivary cystatin secretion from the type of exercise (aerobic or anaerobic), as well as its intensity. Lastly, an *in-vitro* model system was set up using a human submandibular gland cell line to explore the association between cystatin secretion and a β-adrenergic stimulus. Evidence is presented for co-migration of type-S cystatins and cystatin C, suggesting that they may travel in the same packet.

## Materials and methods

### Subjects

Twenty male Brazilian marines (age [median ± S.D.] 28 ± 6 years; 71.6 ± 6.3 kg; 177.0 ± 1.4 cm; 9.6 ± 4.8% body fat; $\dot{V}O_{2\max}$ 56.6 ± 10.7 mL/kg.min) who were also members of the Brazilian Naval Pentathlon Team agreed to participate. Each was injury-free at the time of the studies and instructed not to engage in heavy physical exercise for 48 h prior to the tests, except for scheduled competitions. All served in a military facility and consumed balanced meals arranged by a nutritionist, with water *ad libitum*. Experimental protocols were approved by the Ethics Committee of Clementino Fraga Filho Hospital of the Federal University of Rio de Janeiro (Protocol Number: 030/10).

### Study design for physical exertion

#### Pentathlon competition

**Fig 1:**
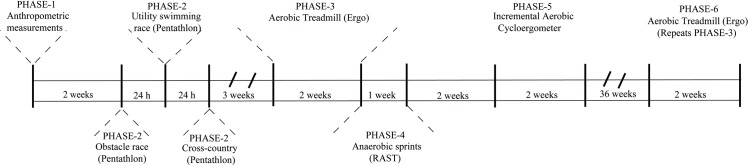
Timeline of events. The study design consisted of 6 distinct phases. In ***Phase 1*,** anthropometric measurements were taken from all participants in the morning (6–8 a.m.) of the first day, 2 weeks before pentathlon competition. In phases 2 to 6, performed between 7 and 10 a.m., pre-exercise (rest) samples of saliva were collected before every test, and post-exercise samples were collected starting 5 min after the test, for exactly one minute. Saliva samples were collected by cotton roll except in phase 6, when saliva was collected by spitting for the purpose of comparing methods. ***Phase 2****)* The three consecutive days of pentathlon competition. Samples were collected before and after the first event of each day. ***Phase 3)*** Starting 3 weeks after the competition, participants performed aerobic physical tests on a treadmill in the morning, and $\dot{V}O_{2\max}$ was recorded. Samples were collected and a maximum of three athletes were evaluated per day. ***Phase 4)*** Two days after phase 3, participants performed anaerobic sprints (RAST) with a maximum of four subjects per day. ***Phase 5)*** Two weeks after phase 4, athletes performed incremental aerobic sprint tests (65%– 95% $\dot{V}O_{2\max}$) on a cycloergometer in the morning with a maximum of two subjects per day. Blood samples for lactate were collected in this phase. ***Phase 6)*** Several months after phase 5, the same athletes repeated phase 3 (treadmill) over a span of **2** weeks.

All physical tests were conducted between 7 and 10 a.m. During a 3-day Naval Pentathlon competition [14], which is a sequence of five intense tasks performed on three consecutive days (Fig 1, phase 2), ranging in duration from about 60 s (life-saving race) to 12 min (cross-country race), pre- and post-exercise samples were collected for the first event of each day: Day 1, an obstacle race on land (~2 min) involving 9 or 10 vertical and horizontal obstacles separated by short sprints over a total of 280–305 m; a life-saving swimming race (~60 s, not included); Day 2, a utility swimming race (~90 s) involving underwater maneuvers and a total of 100 m or 125 m sprints; a seamanship race (~4 min, not included); and Day 3, an amphibious cross-country race of 2500 m (~12 min) with sprints on land of 400, 500, and 800 m as well as 100 m in a boat. During the pentathlon, the distance covered by sprinting varies depending on the task from ~150 meters to 800 meters on land, and ~100 meters in the water. The different tasks also involved lifting, carrying, throwing, climbing, jumping and rowing. Saliva samples were collected after the obstacle race (Day 1), utility swimming race (Day 2) and amphibious cross-country race (Day 3) (Fig 1, phase 2). After the pentathlon (Phase 2 in Fig 1), some of the athletes were assigned to other tasks by their commander and the team was reduced to 12, who performed all the remaining exercises. Physical tests were chosen to elicit maximal anaerobic or aerobic efforts; one test (incremental aerobic) was followed by a post-exercise recovery time course.

#### Aerobic test

$\dot{V}O_{2\max}$ is an essential measure of fitness and also provides a reference value for comparing intensity of effort with other individuals and other types of tests. The $\dot{V}O_{2\max}$ of the 12 participants included in Phases 3 to 6 was determined in Phase 3 using progressively increasing exercise on a treadmill with a duration between 8 and 12 minutes, when the athlete attains his maximum effort. The measurement and analysis of exhaled gases was performed with a VO2000 metabolic analyzer (MedGraphics, United States), which was auto-calibrated before each test. Maximal oxygen consumption was defined as the highest $\dot{V}O_{2}$ obtained over any continuous 30-s time period, provided that the respiratory exchange ratio (RER) was ≥ 1.10 [15]. Saliva was collected before and 5 min after the treadmill test (Fig 1, phase 3). In Phase 6 the treadmill was used by the same subjects but without measuring O_2_ directly, since maximum effort was identified from heart rate.

#### Anaerobic test

RAST (running anaerobic sprint test) was used as an anaerobic test that requires maximum effort. It was applied individually in Phase 4 (Fig 1), outdoors on grass. It consisted of 5 min of a non-strenuous warm-up (stretching and light jogging) followed by 6 sprints of 35 m each at maximum velocity with 10 s rest between sprints [16,17]. Saliva samples were collected before the RAST and 5 min after the last sprint.

#### Incremental aerobic test

An aerobic incremental intensity test consisted of a single session divided into four bouts, at 65%, 75%, 85% and 95% of $\dot{V}O_{2\max}$ on a cycloergometer. Each bout lasted 4 min, with saliva collected at rest and 5, 10 and 15 min post-exercise (Fig 1 –phase 5). Besides the variations in intensity, this test provided information about plasma lactate and about recovery kinetics for total protein, type-S cystatins, and α-amylase.

### Biological samples

Biological samples were collected by trained professionals. Because of the intensity of some of the exercises, athletes required a period of recovery before they could breathe without gasping, a problem also noted by others [18]. Therefore, all of the tests (aerobic as well as anaerobic) were designed so that the first post-exercise saliva samples were taken after a 5-min cool-down period. Unstimulated saliva was collected 5 min prior to a test and then 5 min, 10 min or 15 min after the test according to the particular protocol. In a subset of pre-test samples taken 24 h later, no differences from the original pre-test samples were observed, thereby allowing exercises to be performed on successive days. To restrict evaluations to the composition of saliva being produced at the time of collection, each athlete first rinsed his mouth with deionized water. This was discarded and a 4 x 1 cm cotton dental roll (0.4 g, Cremer Nº.2) (Blumenau, Santa Catarina, Brazil) was inserted under the tongue for 1 min to collect saliva in the absence of mastigation. The saliva containing cotton roll was transferred to the barrel of a 10-ml syringe and the saliva was expelled with the plunger into a sterile tube. Collecting unstimulated saliva as described in our protocol would tend to minimize variability due to interactions between oral stimuli, pH and flow rates [19,20]. As a control for the cotton-roll method, in the final exercise (Phase 6 in Fig 1) the spitting method was also tested [21]. Protease activity was inhibited by adding 1 mM EDTA and 1 mM phenylmethyl sulfonyl fluoride [22], except for samples used to analyze salivary amylase activity (which is inhibited by EDTA). Samples were transported on ice to the laboratory, centrifuged (14,000 x *g* for 10 min at 4°C) and the supernatant transferred to a new tube for storage at -80°C. In phase 5 (see Fig 1), blood (10 ml) was obtained by venous puncture into collection tubes containing sodium fluoride/EDTA and maintained on ice for transport. Plasma was separated by centrifugation (1500 x *g* for 20 min at 4°C) and stored at -80°C for analysis of lactate.

### Biochemical assays

Blood for plasma lactate determinations was collected from an antecubital vein before and after each increment of intensity in phase 5 (Fig 1). Saliva for amylase activity was collected before and after exercise in phases 5 and 6. Lactate and amylase samples were processed in duplicate by spectrophotometry at 340 nm and 660 nm, respectively, according to instructions in commercial kits (Bioclin, Rio de Janeiro, Brazil). Measurements of amylase are presented as a ratio to the resting value, which was designated as 100%. Total protein in saliva was measured by a Bradford assay [23]. In addition, the quantity of protein in individual electrophoretic bands separated by SDS-PAGE was measured by densitometry using colloidal Coomassie blue G staining and an Odyssey infrared scanner [24] in comparison to bands of standard BSA solution run on the same gel.The coefficient of variation for pipetting small volumes of protein followed by staining and scanning the gels was satisfactory for amounts of at least 2 μl, with a coefficient of variation of 6–12% (n = 6). Accuracy was evaluated from signals for ovalbumin added to saliva samples, where average recovery was 103%, with a relative standard deviation of 18%. After background subtraction, standard curves for BSA were linear (R^2^ was 0.96–0.99% in two overlapping ranges, 30–200 ng and 120–800 ng) (n = 6 for each set). Protein from HSG cell lysates and culture medium was quantified by BCA assay kit (Pierce, Rockford, IL, USA).

For Western blots, proteins separated on gradient SDS-PAGE gels were transferred at constant voltage (100 V) to a polyvinylidene difluoride (PVDF) membrane for 90 min at 4°C in transfer buffer (48 mM Tris, pH 8.0; 40 mM glycine and 20% (v/v) methanol). Next, membranes were dried and then rehydrated for an overnight incubation with primary antibodies for cystatin.We were unable to obtain antibodies that were specific to each of the S-type cystatins, which are 90% identical in their primary sequence, so we used a monoclonal antibody for S-type cystatins as a group (SA/SN/S; sc-73884; Santa Cruz Biotechnology, Dallas, TX, USA) diluted 1:1000, and/or a polyclonal antibody against cystatin C (P14) (cat sc 16989; Santa Cruz Biotechnology, Dallas, TX, USA) diluted 1:1000. The next morning, membranes were washed and then incubated 1 h with the appropriate secondary antibodies, diluted 1:10,000: for S-type cystatins, this was goat anti-mouse antibody conjugated with IRDye 800CW (Cat. # 926–32210; LI-COR, Lincoln, NE, USA) and for cystatin C, this was goat anti-rabbit antibody conjugated with IRDye 680RD (Cat. # 925–68071; LI-COR), diluted 1:10.000. After washing, fluorescence was captured and quantified on an Odyssey infrared scanner (LI-COR).

The quantity of cystatin C in cell lysates and culture medium was determined by an ELISA assay, which was available for cystatin C but not cystatins type S (cat # RAB0105; Sigma-Aldrich, St. Louis, MO, USA) as instructed by the manufacturer. Optical density was measured at 450 nm in a VersaMax ELISA Microplate Reader (Molecular Devices, San Jose, CA, USA). The results are presented in ng of cystatin C per μg of total protein.

### Mass spectrometry

For a more detailed confirmation of low-molecular-weight peptides that increase in saliva with exercise, we turned to mass spectrometry. Electrophoresis (1D SDS-PAGE) was performed in a Bio-Rad system (Rio de Janeiro, Brazil). Three micrograms of protein obtained from pooled saliva drawn before and after three pentathlon events (Phase 2 in Fig 1) was separated on a 4–18% gradient gel and stained with colloidal Coomassie blue G. Low-molecular-weight bands at ~15 kDa were excised from the gel, destained (50% (v/v) methanol and 5% (v/v) acetic acid), dehydrated in 100% acetonitrile and dried at room temperature. Proteins in the samples were reduced with 10 mM DTT and alkylated with 100 mM iodoacetamide, at room temperature. Samples were dehydrated again and rehydrated in (NH_4_)_2_CO_3_ (100 mM). Next, proteins were digested with trypsin at a final concentration of 10 ng/μL in (NH_4_)_2_CO_3_ (25 mM). Resulting peptides were extracted with 50% acetonitrile/0.1% trifluoroacetic acid (ACN/TFA) and applied to a C18 ZipTip desalting column of 0.6 μL (EMD-Millipore). The final sample eluted from ZipTip with ACN/TFA was subjected to fractionation and desalting by liquid chromatography in-line to an electrospray ionization quadrupole time-of-flight mass spectrometer (ESI-Q-TOF) as described [16]. Protein sequences were identified by the MASCOT online software (www.matrixscience.com) by comparison to the tandem mass spectra of the National Center for Biotechnology Information proteins and Mass Spectrometry protein sequence database for *Homo sapiens*. One missed cleavage was permitted per peptide and a peptide mass tolerance of ± 0.1 kDa was allowed. Cysteines were assumed to be carbamidomethylated with a variable modification in methionine by oxidation.

### Cell culture, differentiation and treatment

For insight into cystatin secretion in response to adrenergic stimuli at the cellular level, we used an *in-vitro* assay. A human submandibular gland (HSG) cell line [25] was a generous gift from Dr. Marinilce F. Santos (University of São Paulo, Brazil). Cells were grown at sub-confluent densities in tissue-culture bottles using complete medium (Eagle´s Minimum Essential Medium [Sigma-Aldrich], 5% FBS, 2 mM glutamine, 1% [w/v] non-essential amino acids (GIBCO), 100 U/mL penicillin, 100 μg/mL streptomycin, 10 μg/mL gentamicin [Sigma–Aldrich]), at 37 ºC in a humidified atmosphere of 5% CO_2_ and 95% air. For differentiation into acinus-like structures, cells were seeded at a density of 6 x 10^3^ cells/cm^2^ in tissue-culture dishes coated with 10.8 mg/mL GFR Matrigel (Becton-Dickinson, Billerica, MA, USA) and maintained in complete medium for 3 days.

For experimental treatment without differentiation into acini, HSG cells were seeded in 6-well culture plates that received fresh culture medium when the cells reached 80% to 85% confluence. After 24 h, culture medium was supplemented with isoproterenol (IPR) and/or propranolol (PRO) at 100 μM. After 60 min, culture medium was harvested separately and stored at -80 ºC for later analysis. Cells were washed with PBS, collected and stored at -80^°^C. Treatment of cells differentiated into acini was carried out with 100 μM IPR or vehicle for 60 min without a prior medium exchange.

The HSG cell line used in this study originated in Japan in 1981 from irradiated cells of a human submandibular gland. It exhibits various features expected for cells of salivary gland origin, such as formation of acinus-like structures, response to isoproterenol, and synthesis and release of S-type cystatin, typical of submandibular glands [6]. Previously, HSG cells derived from the original sample have been found to be contaminated with HeLa cells in different laboratories [26,27]. To detect HeLa contamination in our HSG cells, we have had short tandem repeat (STR) analysis [27] performed on our sample at 15 loci to verify its genotype. Although the STR signature of the original HSG cells is not known, a match of ≥ 80% to the HeLa cell line at 8 loci considered standard for STR analysis of normal human tissue would be evidence of contamination with HeLa. In fact, not a single locus of 15 tested matched the HeLa pattern, thereby making contamination very unlikely.

### Immunofluorescence

Specific primary antibodies to cystatin C and (as a group) to the 3 salivary type-S cystatins (S, SA and SN) make it possible to identify cystatins in mixtures of cell proteins and quantify them using fluorescent secondary antibodies that are visible in a confocal fluorescence microscope. Stimulated and unstimulated HSG cells in culture were fixed with 2% paraformaldehyde and 0.5% glutaraldehyde in phosphate-buffered saline (PBS) for 5 min, rinsed three times with PBS and then permeabilized with 0.5% Triton-X100 in PBS for 3 min. Next, specimens were blocked with 5% BSA in PBS for 1 hr prior to an overnight incubation with primary antibody, either the monoclonal human anti-mouse cystatin S/SA/SN or rabbit anti-goat cystatin C at a dilution of 1:400. The next day, specimens were washed three times in PBS with 0.1% Tween 20 for 5 min each followed by incubation for 2 h with a 1:400 dilution of a rabbit anti-goat secondary antibody labeled with Alexa 594 (Cat# A27016; ThermoFisher, Grand Island, NY, USA) for cystatin C and a donkey anti-mouse secondary antibody labeled with Alexa 488 (Cat# R37114; ThermoFisher) for cystatins type S. DNA was detected directly with DAPI (4',6- diamidino-2-phenylindole, ThermoFisher). Prior to mounting, coverslips were washed an additional three times in PBS-Tween 20 for 5 min each.

### Microscopy and image quantification for cell cultures

For quantification of the cystatin distributions following an adrenergic stimulus, raw fluorescence images were obtained from multiple fields of HSG cell cultures that were fixed, permeabilized and labelled as described in the previous paragraph and examined with an LSM510 confocal microscope (Zeiss, Munich, Germany). Excitation of Alexa 594 was at 590 nm and emission at 620 nm, while Alexa 488 was excited at 500 nm and emission collected at 520 nm. To determine the total surface area of an acinus-like structure, each of which contained a different number of individual cells, the software ImageJ v.1.51h [28] was used to draw an outline of its perimeter. Next, two regions of interest were defined: a peripheral (P) ring that began at the perimeter and whose thickness was adjusted to the interior of the cell grouping to cover ~37% of the total area; and an internal (I) circle in the center of the same structure that covered ~3.7% of the total area. Within each region, the total corrected area of fluorescence (TCAF) was calculated from the measured integrated fluorescence signal by subtracting the background fluorescence that was measured in the same field but with no cells inside it, and covering the same area as the regions of interest. By calculating a ratio of the TCAF of a circular peripheral ring, P, to the TCAF of its corresponding interior circle, I, the change in the fluorescence from the center to the periphery could be observed as a value greater than calculated from the controls. Furthermore, by measuring an equivalent percentage of the total area of an individual structure, the contribution of the size differences between individual acinus-like structures was factored out.

### Statistical analysis

Data are shown as the median ± S.D, except as noted. Differences between sample groups were compared by a Mann-Whitney Rank Sum Test or one-way ANOVA, as indicated in the figures. Statistical significance was defined as p< 0.05.

## Results

### Mass spectrometry

**Fig 2:**
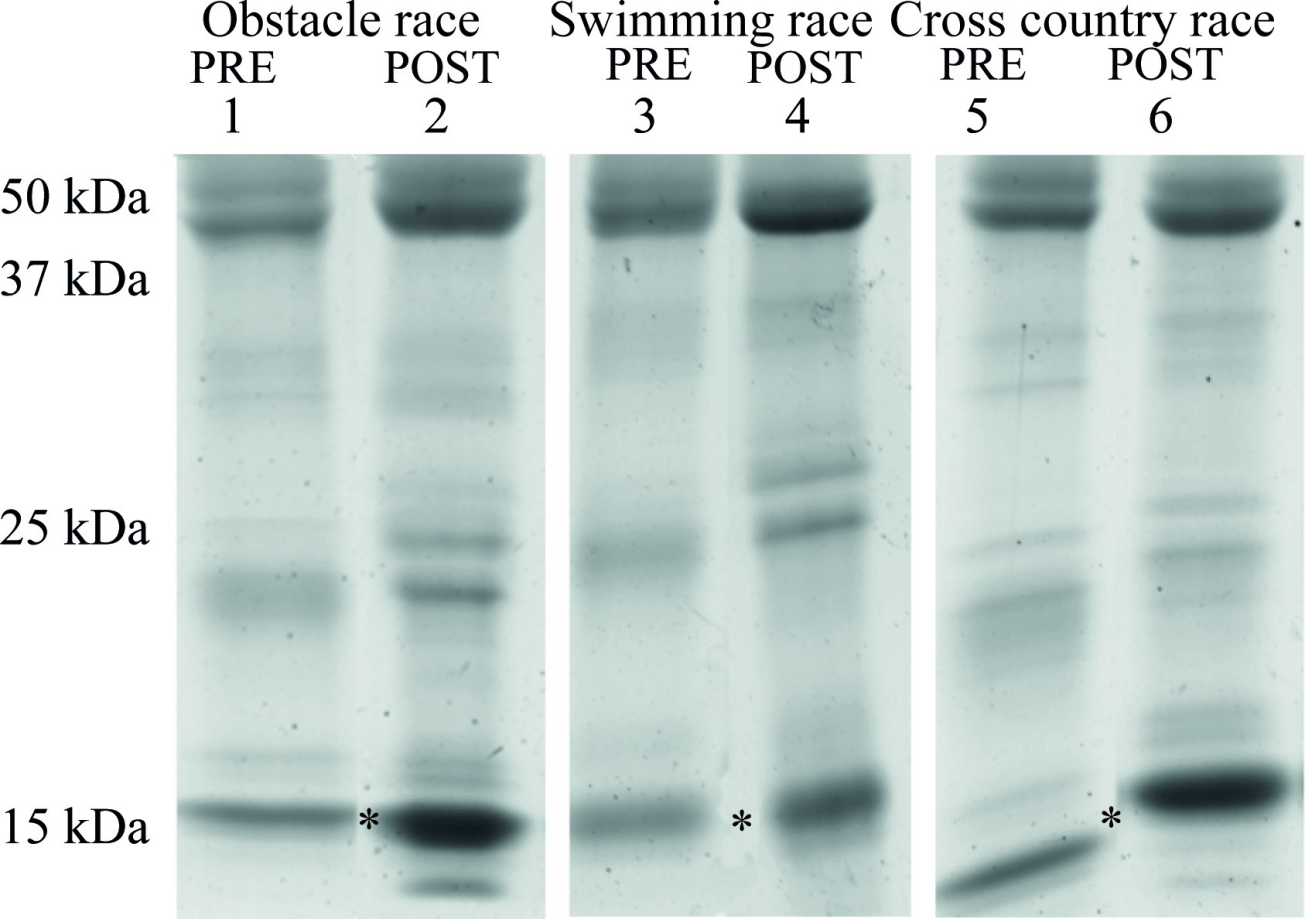
SDS-PAGE analysis of human saliva. Samples of saliva from the 20 participants in a three-day pentathlon competition (phase 2 in Fig 1) were pooled from before (*pre*) and after (*post*) the first competitive event on each day: Day 1, obstacle race, Day 2, utility swimming race and Day 3, amphibious cross-country race. The asterisks at ~15 kDa indicate the low-molecular-weight bands of interest that were excised and analyzed by mass spectrometry in Table 1.

Initially, the protein pattern in saliva was compared between six pooled samples obtained from 20 athletes before and after the first event each day of a three-day pentathlon competition (Phase 2 in Fig 1). Since the competition included events that required different types of physical exertion, this approach also permitted a preliminary evaluation of the effect of the type of physical activity on protein profile differences. The gradient SDS-PAGE gels in Fig 2 show the protein pattern in the pools of saliva collected, with each panel representing the before and after of a specific task: an obstacle race; a utility swimming race; and an amphibious cross-country race.

Regardless of the day and the task, the presence of a low-molecular-weight protein was consistently increased in the post-task samples (Fig 2). To identify this protein, the region encompassing the band was excised from a gel and prepared for analysis by mass spectrometry following trypsinization. A comparison of the resulting peptide spectra to the NCBI database for human proteins by MASCOT identified 26 fragments of 17 individual proteins. In the region of the gel excised (12–17 kDa), the relevant peptides were derived primarily from proteins of the cystatin family. The data in Table 1 are organized by status of samples (pre- or post-exercise) and gel lane, with a requirement of 7% coverage or better for the parent protein.

**Table 1:** Proteins identified by mass spectrometry.

Nº	Status	Gel Lane	Protein Identified	NCBI	% Cov^†^	Score	Peptides	Mass (Da)
1	Pre	1	Cystatin S precursor	NP_001890	12	89	1	16489
2	Pre	1	Cystatin SN–partial peptide, 35 aa	AAB20561	51	73	1	4131
3	Post	2	Cystatin SN precursor	NP_001889	12	69	1	16605
4	Post	2	Cystatin S acidic isoform–partial peptide, 35 aa	AAB20560	51	57	1	4022
5	Post	2	Cystatin SA-III	AAB19889	80	275	8	14409
6	Pre	3	Keratin 1	AFA52006	32	583	16	66197
7	Pre	3	Keratin, type I cytoskeletal 9	NP_000217	24	419	10	62255
8	Post	4	Cystatin-SN precursor	NP_001889	60	473	10	16605
9	Post	4	Cystatin-SA precursor	NP_001313	34	117	3	16719
10	Post	4	Unnamed protein product	BAG36698	34	682	17	66151
11	Post	4	Cystatin S acidic isoform–pkakanisartial peptide, 35 aa	AAB20560	51	57	1	4022
12	Pre	5	Cystatin S precursor	NP_001890	18	139	2	16489
13	Pre	5	Keratin, type II cytoskeletal 2 epidermal	NP_000414	17	105	5	65678
14	Pre	5	Keratin, type I cytoskeletal 10	NP_000412	15	205	6	58994
15	Pre	5	Cystatin SN–partial peptide, 35 aa	AAB20561	51	57	1	4131
16	Pre	5	Thioredoxin isoform 1	NP_003320	12	27	1	12015
17	Pre	5	Alpha-amylase 1 precursor	NP_004029	8	60	2	58415
18	Post	6	Cystatin B	NP_000091	12	31	1	11190
19	Post	6	Cystatin D	CAA49838	20	78	2	16351
20	Post	6	Cystatin C precursor	NP_000090	7	39	1	16017
21	Post	6	Cystatin SN precursor	NP_001889	48	285	4	16605
22	Post	6	Cystatin SN precursor	NP_001889	36	170	3	16605
23	Post	6	Keratin, type II cytoskeletal 1	NP_006112	28	869	12	66170
24	Post	6	Cystatin S precursor	NP_001890	25	127	2	16489
25	Post	6	Cystatin SA precursor	NP_001313	23	143	2	16719
26	Post	6	Keratin, type I cytoskeletal 9	NP_000217	27	424	7	62255

The 15–17 kDa bands from saliva purified by SDS-PAGE of 6 pools of athletes´ prior to (Pre) or following (Post) maximal physical exertion. Note that Mass in last column refers to the protein identified as the *origin* of the peptide(s) analyzed, which were all collected near 15 kDa and trypsinized for analysis (Fig 2).

^**†**^Percent coverage of identified protein by peptide(s) analyzed.

One or two peptides from cystatin C and α-amylase were present, but provided only 7–8% coverage of the protein. From lane 6 of Fig 2, most peptides were from cystatin-SN (cysSN), along with others from cystatin-SA (cysSA). Cystatin-B, cystatin-C (cysC) and cystatin-D were also present. The bands excised from lanes 2 and 4 were principally composed of cysSA and cysSN. The other peptides appeared to be contaminants derived from higher-molecular-weight proteins such as α-amylase and an assortment of keratins.

### Physical tests and measurements of protein concentration

The behavior of the cystatins identified as the principal low-molecular-weight salivary proteins that increased in intensity following different types of physical exertion was evaluated further following physical exercises of prescribed intensity and defined type.

**Fig 3:**
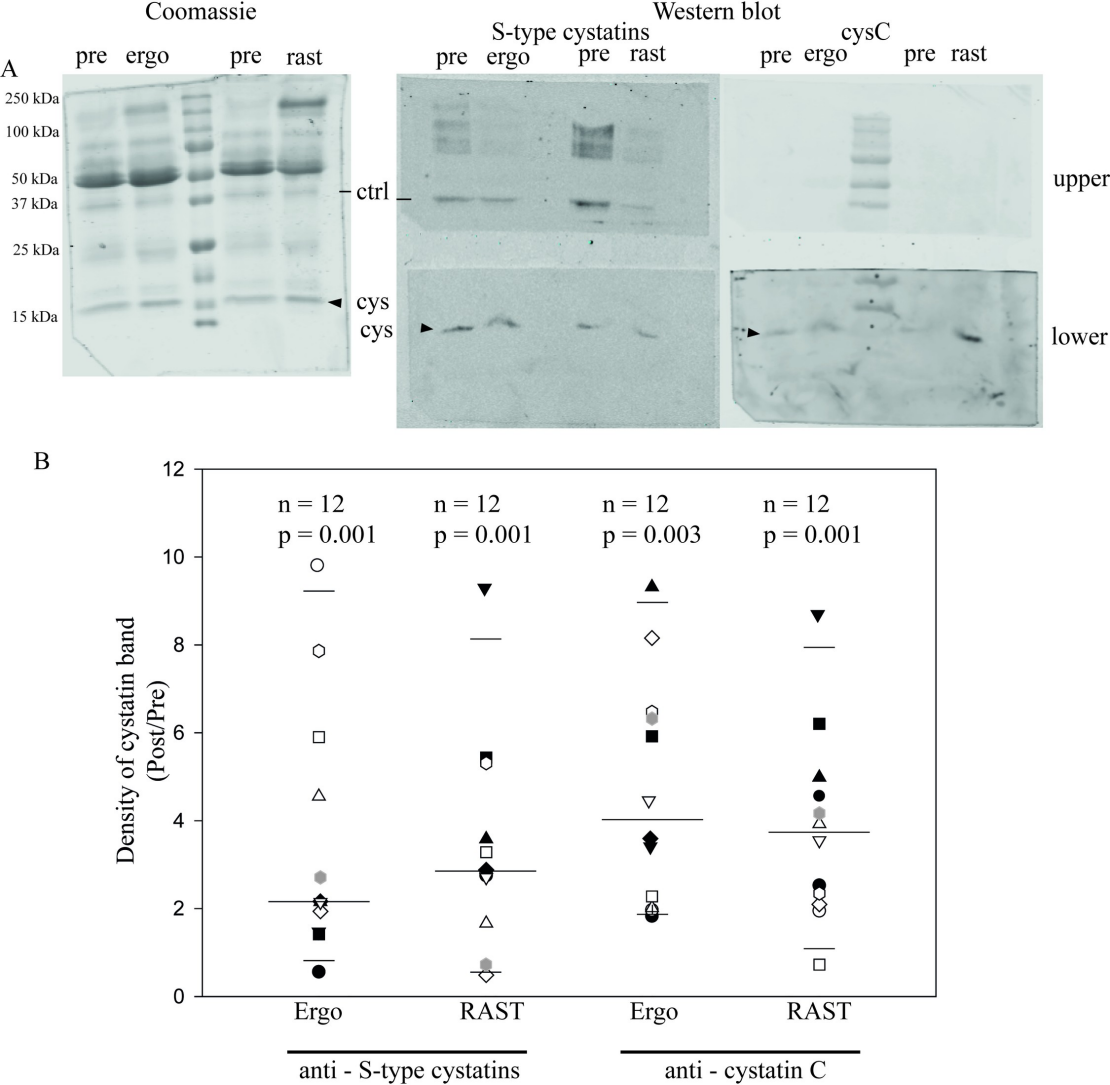
Maximum exertion increased salivary cystatin secretion. Saliva was collected from participants before (*pre*) and after performing on separate days either an aerobic exercise (treadmill ergospirometry to $\dot{V}O_{2\max}$) (*Ergo*), or an anaerobic exercise, *RAST* (Phases 3 and 4 of Fig 1). In panel A, exogenous ovalbumin (*ctrl*) was added to each sample as a loading control. Shown is a representative SDS-PAGE (left) stained with colloidal Coomassie blue G and the corresponding Western blot (right) for samples from a single athlete. The Western blots in the *upper* panels were probed for ovalbumin (42.7 kDa) and those in the *lower* panels were probed for cystatins (*cys*, arrowheads), either type-S (S, SA and SN) or cystatin C (*cysC*). Panel B shows a box plot with the median, 10^th^ and 95^th^ percentiles of the quantified intensity ratios for type-S cystatins and cystatin C measured from Western blots for each saliva sample from each participant. Each data point represents the ratio of post- to pre-exercise intensities in Western blots for that band from each individual. The number of subjects and the p-value for this ratio from a Mann-Whitney Rank Sum Test are shown above each column.

First we compared saliva samples from aerobic exercise on a treadmill to $\dot{V}O_{2\max}$ (Ergo, phase 3, Fig 1) and samples obtained following exhaustive sprints for anaerobic exercise (RAST, phase 4 in Fig 1). Western blots were performed on individual saliva samples collected pre- and post-exercise using an antibody against S-type cystatins (S, SA, and SN) and another against cystatin C. A band of approximately 16 kDa was detected by antibodies to S-type cystatins and cystatin C, as expected (Fig 3A, center and right). Quantification of Western blot intensities showed that the mean secretion of both cystatins was increased by both aerobic and anaerobic exercise (Fig 3B).

There was no statistically significant difference in cystatin ratios Post/Pre between the two types of exercise or between the two types of cystatins. In the box plot, the median in each column is indicated by the longest horizontal line; the entire range of individual values is shown using a different symbol for each athlete. The elevation in the amount of cystatins detected in saliva after exercise varied in each experimental group. Although there was a significant increase from the resting value (= 1.0) in all four columns, four individuals were exceptions to the trend and did not show an increase in cystatin secretion after specific physical exercises: one in cystatins type S-Ergo; two in cystatins type S-RAST; and one in cystatin C-RAST (Fig 3B). For cystatins type S-Ergo, the median value of the ratio comparing cystatin post- vs pre-exercise was 2.1 ± 2.8 fold; for cystatins type S-RAST, 2.8 ± 2.4 fold; for cystatin C-Ergo, 4.0 ± 2.6 fold; and for cystatin C-RAST 3.7 ± 2.1 fold (median ± SD, n = 12).

Since an increase in cystatin concentration could occur if there were a generalized decrease in salivary volume (for example by evaporation or a lower flow rate), we also measured volume and total protein concentration. Table 2 (column 7) shows that the volume of saliva collected in one minute was routinely lower post-exercise compared to the pre-exercise value, but even for the most strenuous type of exercise (RAST) the decrease in volume (by 43% on average) was not enough to account for the increase in cystatin concentration (from a ratio of 1.0 at rest to a ratio of 2.8 for cystatins type S and 3.7 for cystatin C) in Fig 3. Thus the observed increases in cystatins post-exercise cannot be attributed to changes in the salivary flow rate.

**Table 2:** Total protein concentration and volume of saliva in different events pre- and post-exercise.

Phase (Fig 1)	Test	Intensity	Method	Volume (mL)			Total protein (μg/mL)		
				Pre	Post	Post/Pre	Pre	Post	Post/Pre
5	Aerobic (Incre-mental)	95% VO_2max_	Cotton Roll	0.52±0.05	0.49±0.05	0.94±0.09	542.7±185.4	907.8±116.9	1.68±0.81
6	Aerobic (Ergo)	Max	Spit	0.69±0.11	0.48±0.09	0.77±0.12	1305.2±260.1	1871.0±270.8	1.40±0.18
3	Aerobic (Ergo)	Max	Cotton roll	0.76±0.14	0.53±0.06	0.66±0.11	473.8±81.2	883.4±102.4	1.88±0.42
4	Anaerobic (RAST)	Max	Cotton roll	0.62±0.06	0.35±0.08	0.58±0.17	613.0±99.3	1188.4±211.5	2.01±0.43

Data show median ± s.d. (n = 12). Saliva was collected for 1 min, starting 5 min after exercise and rinse.

On the other hand, tests executed by the athletes at maximum or near-maximum effort raised the overall protein concentration at 5 min (Table 2), and there were similar increases in cystatin and total protein (*cf*. ratios Post/Pre for protein in Table 2 and type-S cystatins in Fig 3). This could mean that exercise causes a generalized increase in release of salivary proteins, including cystatins, rather than specific changes such as those reported for lysozyme, IgA and other peptides of the immune system [29].

### Incremental load test

**Fig 4:**
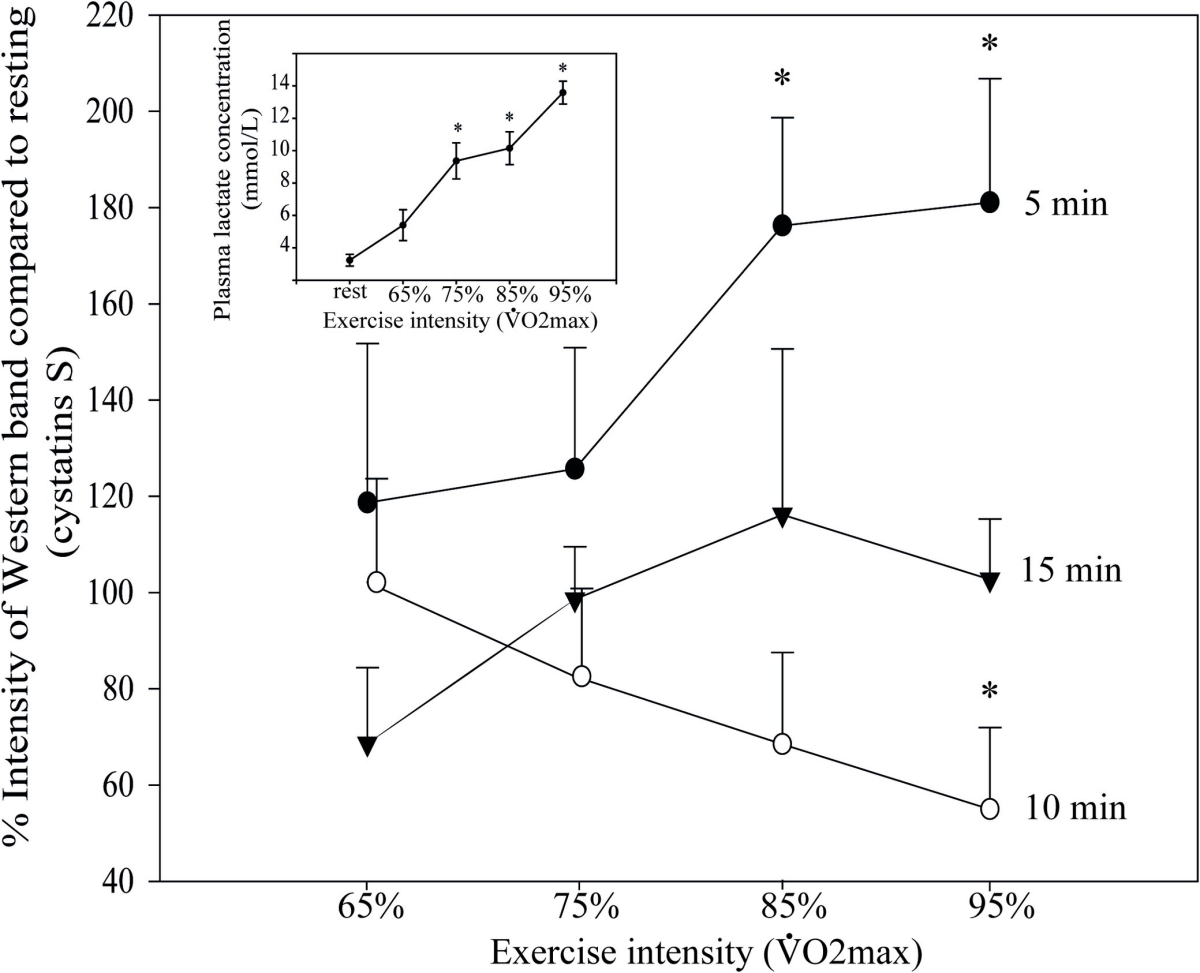
Cystatin secretion in saliva is workload-dependent. Cystatin S/SA/SN secretion in saliva was measured at rest and 5 min (black circles), 10 min (white circles) and 15 min (triangles) after cycling for 4 min at the indicated workload intensity (Phase 5 in Fig 1). *Inset* shows plasma lactate levels at rest and immediately post exercise at each stage(n = 12, *p<0.05 with respect to resting value). Statistics were performed by Kruskal-Wallis one-way analysis of variance on ranks.

To evaluate the influence of exercise intensity and the time course of post-exercise recovery we examined cystatins S/SA/SN in athletes performing an aerobic cycloergometer protocol at four different load increments that lasted 4 min and reached 65, 75, 85 and 95% of $\dot{V}O_{2\max}$ (Phase 5 in Fig 1). Saliva samples were collected at rest and at 5, 10 and 15 min after each load, and the amount of S-type cystatins (as a group) present in saliva was determined by quantitative Western blots. The upper curve in Fig 4 (main panel) shows that there was a significant increase at 5 min, the first post-exercise time point, but only for the two higher loads. These increases were 76.2% ± 22.4 at 85% $\dot{V}O_{2\max}$ and 81.0% ± 25.7 at 95% $\dot{V}O_{2\max}$ (p = 0.012, n = 12). However, the increases were transient: ten minutes after the effort at 95% $\dot{V}O_{2\max}$, the concentration of cystatins S/SA/SN had already fallen to 54.9% ± 16.9% of rest level (p = 0.012, n = 12). By 15 min post-exercise, the levels of cystatin showed no significant difference from the initial rest values (100% on the ordinate).

In the experiment of Fig 4, the intensity of muscle work was confirmed by measuring plasma lactate (inset). Many different laboratories have demonstrated that lactate and catecholamines increase in parallel during intense exercise [30,31]. In a protocol similar to ours and performed by similarly trained athletes ($\dot{V}O_{2\max}$ 58 ml/kg.min), the concentration of lactate increased from 1 mM to 12–13 mM and the epinephrine and norepinephrine reached 10 and 34 pmol/mL, compared to resting values of 0.3 and 2.5 pmol/mL [30]. Thus we have indirect evidence that the exercise intensity employed here produced large increases in plasma catecholamines as a driving force for the increase in cystatins [31,32].

**Fig 5:**
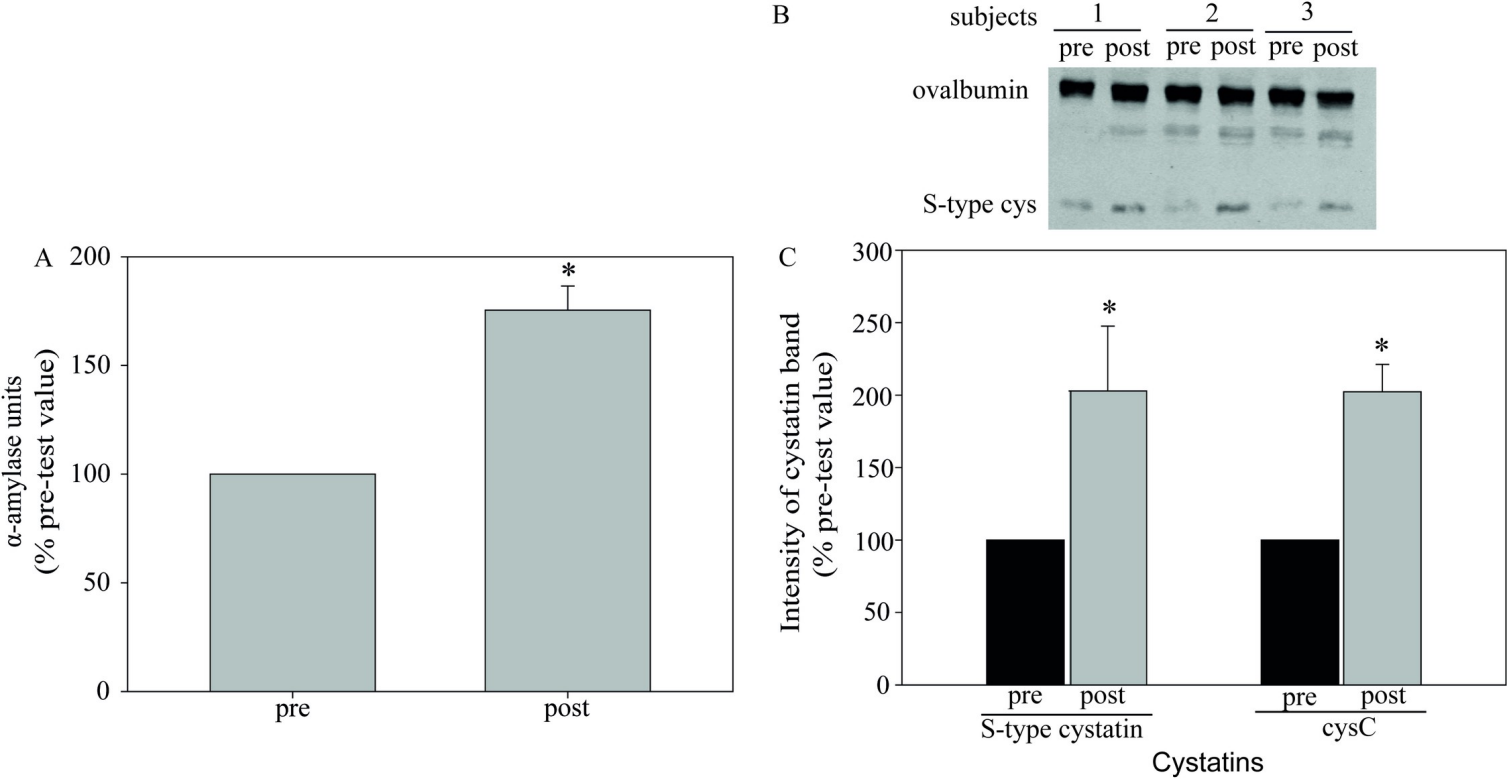
Post-exercise secretion of cystatins to saliva accompanies the increase of salivary amylase activity. Saliva was collected from participants before and after ergospirometry to $\dot{V}O_{2\max}$ on a treadmill (Phase 6 in Fig 1). Panel A shows the relative values of salivary amylase activity post-test compared to pre-test (100%). Panel B is a representative Western blot of S-type cystatins (*cys*) from 3 of the volunteers tested. Cystatins are present in the lower bands; ovalbumin in the upper bands was used as a loading control. Panel C, Densitometry of the cystatin bands in Western blots of saliva from 12 athletes. Intensity of each cystatin band was normalized to the immunoblotted ovalbumin band in the same lane, and then calculated in relation to resting value as 100%.*p = 0.001 compared to pre-test value, Mann-Whitney rank sum test.

A second indication of a strong sympathetic stimulus was provided by measurements of salivary α-amylase, a recognized sympathetic response component. Fig 5A compares amylase secretion before and after exercising to $\dot{V}O_{2\max}$ on a treadmill (Phase 6 in Fig 1). Salivary amylase activity increased by 75% while both cystatins doubled in amount (Fig 5B and 5C).

**Fig 6:**
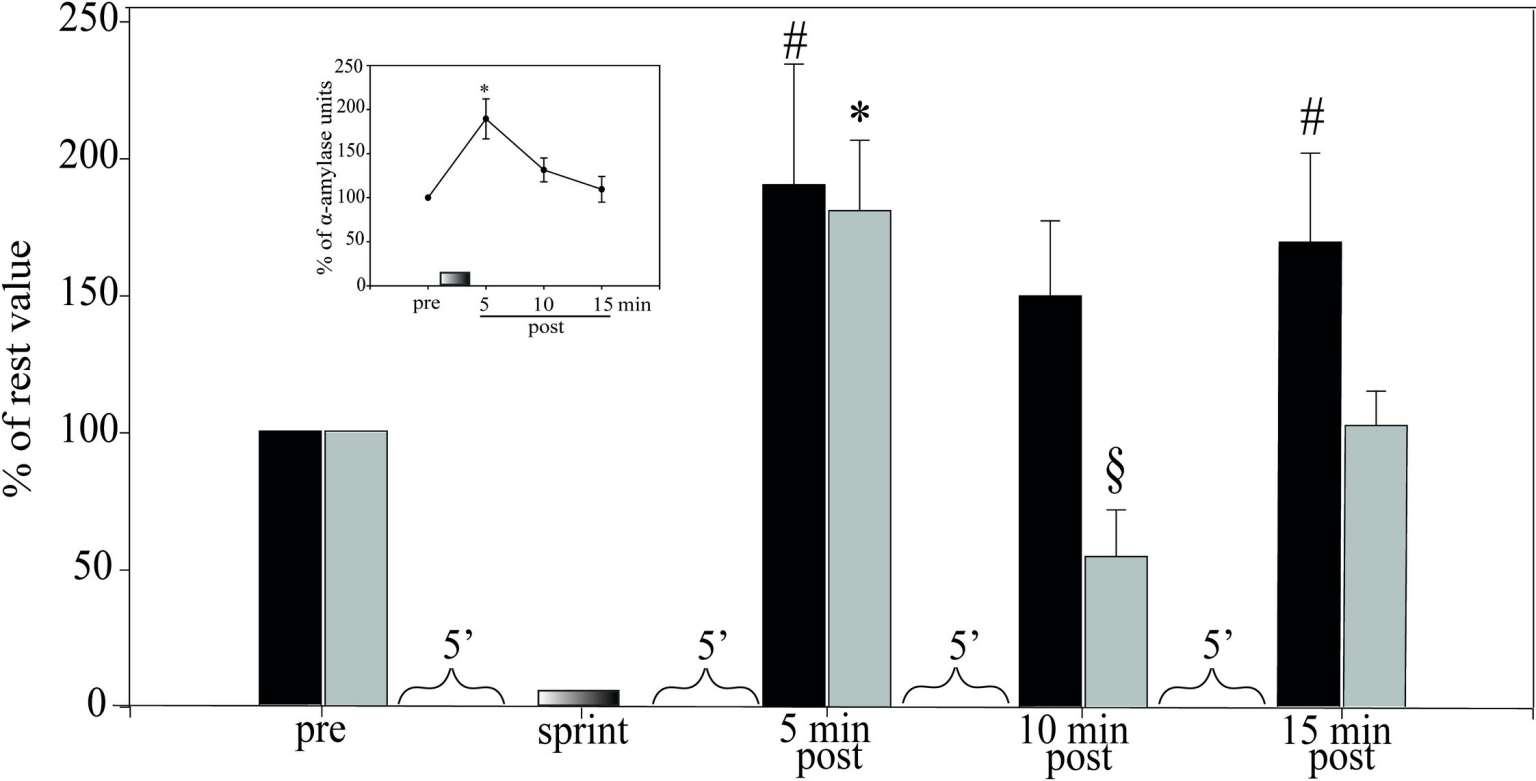
Increased secretion of cystatin in response to exercise is transient. Last stage of incremental aerobic sprints of 4 min each on a cycloergometer (Phase 5, Fig 1). Saliva was collected at rest (*pre*) and during recovery (*post*) 5, 10 and 15 min after reaching 95% $\dot{V}O_{2\max}$ and analyzed for total protein (black bars), type-S cystatins (gray bars) and amylase activity (*inset*). The Y axis shows these data relative to rest as 100%. Sprints at 65%, 75% and 85% $\dot{V}O_{2\max}$ following first sample are shown in Fig 4. # indicates a significant increase compared to total protein at rest (*pre*)(p = 0.012, n = 12). * indicates a significant increase of type-S cystatin at 5 min and § a significant decrease at 10 min compared to resting value (p = 0.012, n = 12). *Inset* shows the α-amylase units in saliva over time with *p = 0.001 compared to resting value, n = 12. ANOVA, Dunn’s method.

For the time course of recovery for salivary amylase, total protein and S-type cystatins after exercise, we return to the incremental aerobic test on a cycloergometer following its last stage (95% of $\dot{V}O_{2\max}$). Here the amylase increased to 189.5±22.7% of the resting value at 5 min post-exercise (Fig 6 *inset*), then fell slowly over 15 minutes. The main panel shows that total protein and type-S cystatins were also maximal at 5 min, but cystatins fell immediately to 54.9% of the resting value at 10 min before recovering, while total protein stayed up (Fig 6) Thus the time course of recovery for the cystatins differs from that of the other proteins and from amylase as well. The picture that emerges here is one where amylase and type-S cystatins might share an initial response/burst with other proteins, but recover along divergent pathways.

### Propranolol, an adrenergic antagonist, blunts cystatin C production in a cell line from human submandibular gland

In salivary glands, agonists of the β-adrenergic system bind to β-adrenergic receptors that activate adenylyl cyclase to generate intracellular cAMP, which in turn activates cAMP-dependent protein kinase (PKA) to increase exocytosis from acinar cells [33,34,35]. The data described so far suggest a response of cystatin secretion to an elevated sympathetic signal provided by physical exercise. In the next experiments, an *in-vitro* model system was set up to evaluate the relationship between a well-defined adrenergic signal and cystatin secretion at the cellular level. A detailed analysis of the secretory pathway for cystatins was beyond the scope of this study, but we were able to obtain and propagate a line of immortalized cells from human submandibular gland (HSG) for preliminary experiments.

**Fig 7:**
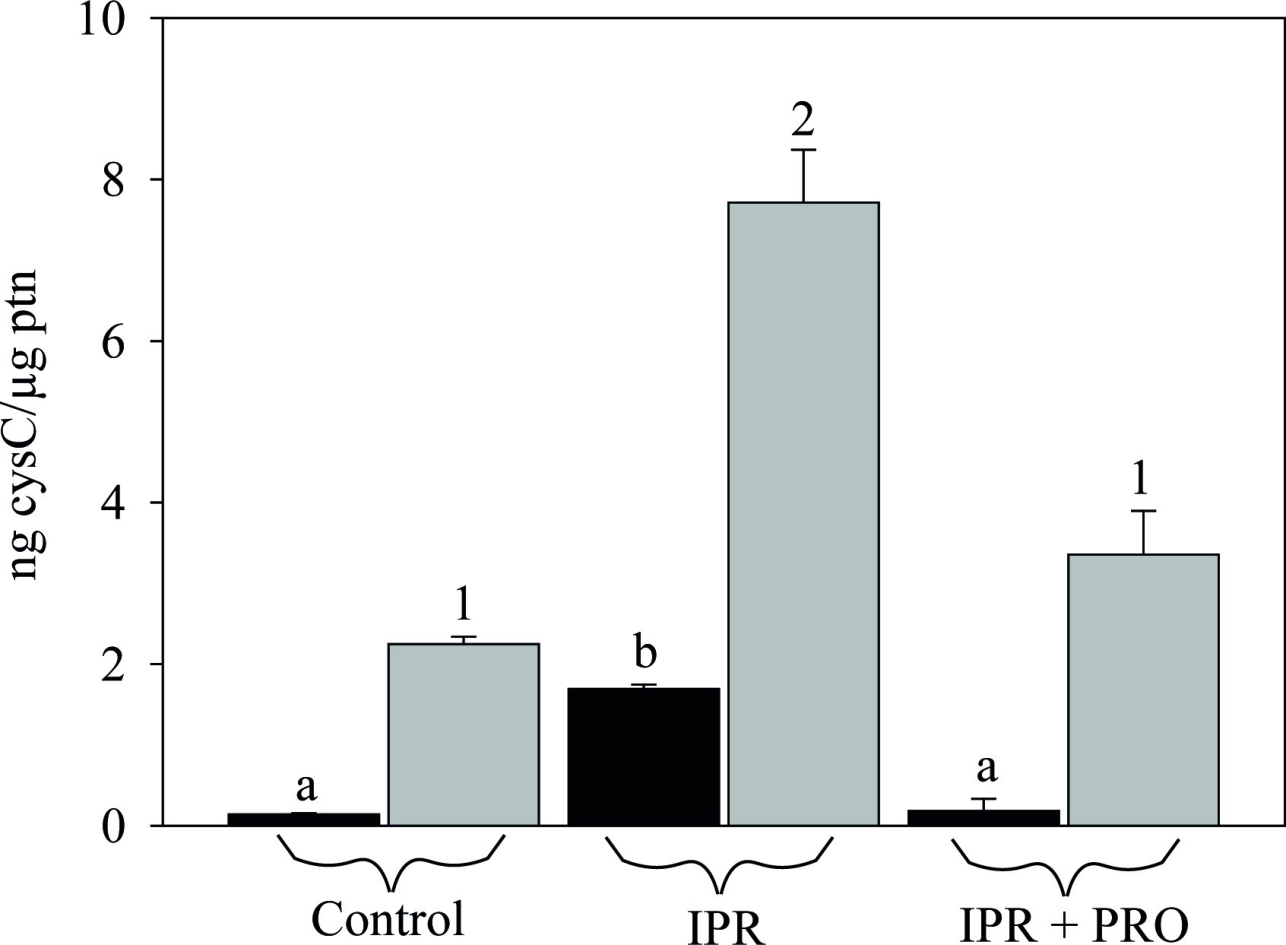
Isoproterenol increases and propranolol inhibits expression and secretion of cystatin C. Elisa assay reveals enhanced cystatin C expression and secretion in cultured HSG cells incubated with IPR (100 μM, 60 min). Propranolol (100 μM, 60 min) decreases cystatin C production. *Gray bars* represent endogenous cysC and *black bars* represent cysC secreted to the medium. Different letters indicate statistical differences in secretion to the medium, p<0.001. Different numbers indicate statistical differences in cellular content, p<0.001. One-way ANOVA (Holm-Sidak method). Bars indicate the mean±s.e. of 3 independent experiments performed in duplicate.

An ELISA assay confirmed the response to a β-adrenergic stimulus in HSG cells plated on plastic. The secretion of cystatin C to the culture medium increased 11 fold with the addition of isoproterenol (IPR) (Fig 7; from 0.14 to 1.7 ng cystatin C/μg protein). The β-adrenergic antagonist propranolol (PRO) competes with isoproterenol at the β-adrenergic receptor, which blocks the activation cascade to PKA [33]. Here, we found that propranolol markedly reduced cystatin C secretion into the culture medium (from 1.7 ng/μg total protein with IPR to 0.18 ng/μg total protein with IPR+PRO). Like secretion, the expression of cystatin C was positively affected by IPR treatment, resulting in an increase to ~3.4 times the control (from 2.25 to 7.71 ng cystatin C/ μg protein) that was blocked when PRO was present at the same time (Fig 7, last column). This is consistent with data showing that propranolol inhibits α-amylase secretion in a parotid epithelial cell preparation [36].

### Adrenergic agonist alters the distribution of cystatins type-S and cystatin C in cultured human submandibular gland acini

**Fig 8:**
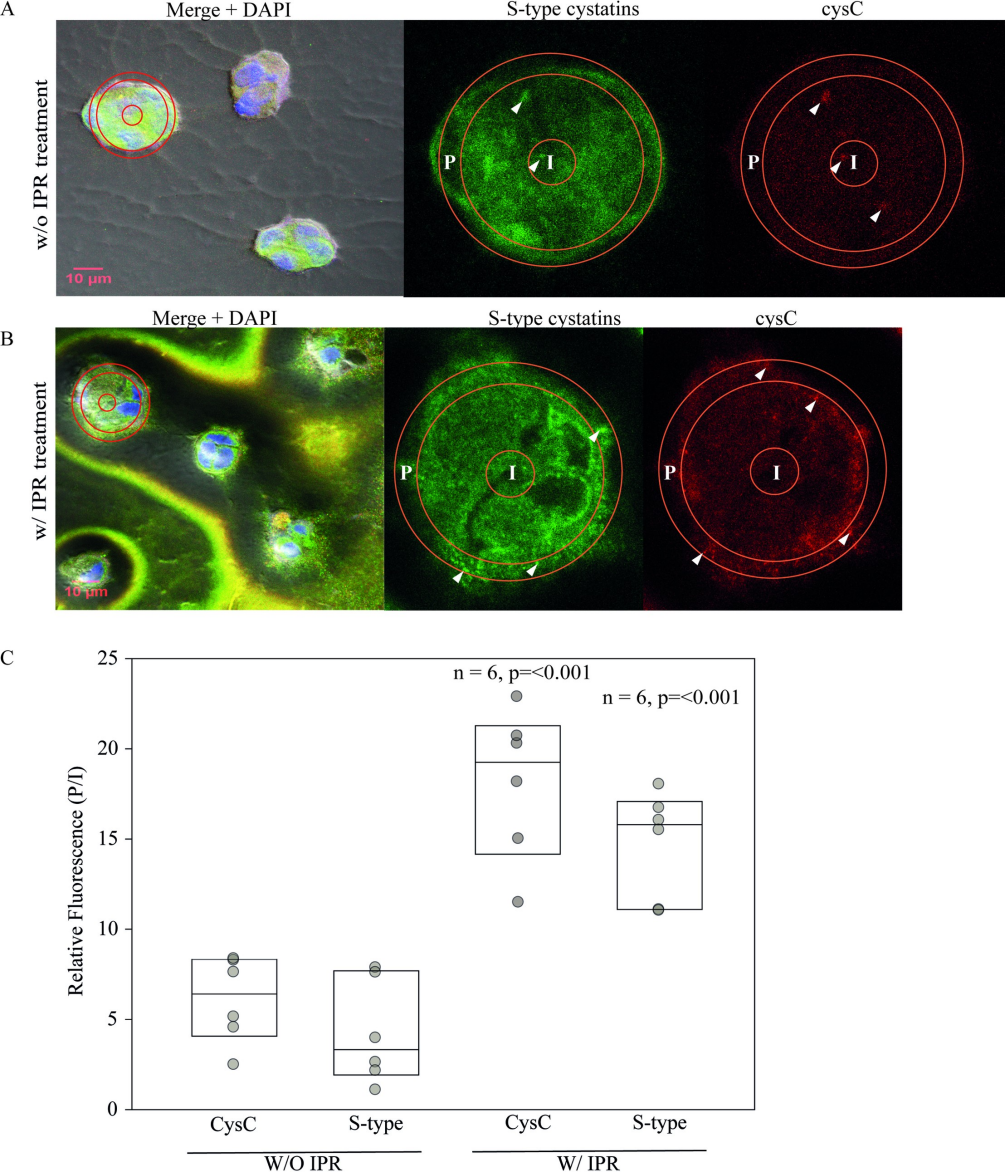
Isoproterenol stimulates cystatin transport to the border of acinar structures. Human submandibular gland cells grown in Matrigel-coated plates spontaneously form acinar-like structures from multiple cells. The merged images at *left* in Panels A (untreated) and B (treated with 100 μM isoproterenol) show representative acinus-like structures formed in Matrigel with nuclei stained with DAPI (blue) together with the intracellular distribution of S-type cystatins (green) and cystatin-C (red). In Panel B, with IPR (*left*), a substantial quantity of both cystatins has been expelled to the medium. The structures at *left* outlined with red rings are digitally enlarged (3.3x) in the two images at *right* for S-type cystatins and cystatin-C. Arrowheads indicate cystatin aggregates labeled in the acini. The red rings outline regions of interest at the periphery (P) and in the center (I) of the acinus-like structures where fluorescent signals were measured either without treatment or after 1 h with 100 μM isoproterenol. Panel C shows the P/I ratios either with or without IPR treatment for each antibody (cysC and S-type). The increase in this ratio with IPR shows the shift in intensity of fluorescence towards the periphery as a response to treatment (box plots show median, 25th and 75th percentiles). P <0.001 in both experiments (n = 6) from a Mann-Whitney Rank Sum Test comparing stimulated and unstimulated P/I ratios.

When immortalized HSG cells are cultured on an appropriate organic substrate instead of unadorned plastic, they spontaneously differentiate into acinus-like structures that assume morphological and functional features of submandibular glands, including the expression of cystatin SN [37]. In the next experiment, we evaluated the endogenous distribution of cystatin C and S-type cystatins in acinus-like structures treated with 100 μM IPR. Human submandibular gland cells were cultured on the matrix Matrigel, where they differentiated over 3 days into asymmetrical acinus-like structures that were 20–30 μm in diameter (Fig 8A, *left*). Staining of the nuclei by DAPI (blue) shows the presence of multiple cells in each acinus-like cluster. The immunofluorescent localization of cystatin C (red) and S-type cystatins (green) in the non-stimulated state (upper panels) reveals a generalized intracellular distribution, and the yellow of the merged images within the cell clusters at left in panel A is consistent with co-localization of the two cystatins in the same intracellular compartments.

With the addition of 100 μM isoproterenol (Fig 8B, *left*), this suggestion of co-localization was reduced but there was a substantial release of cystatin C and S-type cystatins to the medium, now visible in yellow in the merged image outside as well as inside the acinus-like structures following fixation and exposure to fluorescent antibodies. At a higher magnification (Fig 8B, *center* and *right* panels), a comparison of the distribution of the cystatins between the center and periphery of each multicellular acinus was carried out by measuring the fluorescence in an interior circle (I) and a peripheral ring (P), which were used to calculate a fluorescence ratio (P/I). In the absence of IPR (panel A and panel C, *left*), both cystatins showed a preferential distribution towards the interior of acinus-like structures. For cystatin C, this ratio (P/I) increased from 6.4 ± 2.4 without IPR to 19.3 ± 4.2 with the addition of IPR; and for S-type cystatins, P/I increased from 3.3 ± 2.9 fold without IPR to 15.8 ± 3.0 with IPR (median ± S.D.).

## Discussion

Of the more than 2,000 proteins identified in human saliva, only a subset of 200 to 300 are secreted by the salivary glands [34]. The general transport of secretory granules in cells of the salivary glands can be increased by β-adrenergic signaling [38] which is activated by physical exercise through the innervaton by sympathetic neurons [39]. The amount of α-amylase stands out, and can reach ~ 30% of the total protein secreted to whole saliva in response to maximum effort [38].

To our knowledge, our study is the first description of cystatins as proteins whose concentration in saliva responds to exercise. Our measurements of salivary amylase and plasma lactate in trained athletes are consistent with the premise of exercise with a high β-adrenergic component. While cystatin C is produced in many different organs, the S-type cystatins are mainly expressed in salivary glands. The S-type cystatins are of particular interest since they originate primarily from the submandibular glands and not, like amylase, from the parotids. In our experiments the cystatins appeared as a low-molecular-weight protein band in whole saliva that showed a clear increase in intensity after athletes performed a variety of strenuous physical activities. Mass spectrometry of the excised band identified two components of the cystatin-2 family, cystatin C and cystatins type S, as proteins sub-acutely modulated by exercise. Commercial antibodies were available for cystatin C and also (as a group) for cystatins type S. Using these antibodies in quantitative Western blots, we found that both aerobic and anaerobic exercises performed at or near maximal intensity led to an increased presence of both cystatins in saliva. Both showed statistically significant changes when analyzing the 12 athletes as a group.

In recent decades, saliva has gained acceptance as a readily accessible body fluid that provides indirect access to physiological changes accompanying exercise, as well as oral health at rest and the monitoring of therapeutic drugs circulating in the blood [40]. This study did not begin with the expectation of identifying the physiological function of enhanced cystatin secretion during exercise. Rather, the objective was (1) to look for associations with different exercise parameters (intensity, duration, aerobic, anaerobic etc) in order to identify suitable conditions for studying the secretion, and (2) to test a model for investigating the mechanism (and regulation) of cystatin secretion *in vitro*.

Nevertheless, it is interesting to examine the question of what purpose might be served by increased cystatin secretion during exercise; in the last decade an increasing number of other salivary peptides and proteins have been investigated in this context. Both cystatins increase in some pathological states and decrease in others [6,18,41,42]. Cystatins are natural inhibitors of cysteine proteases such as papain and the lysosomal cathepsins, and an obvious question is whether this inhibitory activity is related to their role in saliva [43]. Indeed, cystatin SN is upregulated in humans to combat allergic rhinitis in the nasal cavity, relying on its ability to inhibit protease allergens from pollen [42]. On the other hand, Ganeshnarayan et al [44] identify two common species of oral bacteria that are killed by salivary cystatin SA, but the authors provide evidence against any requirement for protease inhibition in this role. With the multiplicity of different bacterial species in the oral cavity, a case can be made that one function of cystatins in saliva is to inhibit bacterial adhesion inside the mouth. An example is cystatin SA, which blocks the binding of bacteria to buccal epithelial cells [44]. Cystatins can also inhibit bacterial growth without inhibiting protease activity [45].

A potential link between cystatins and exercise appears in recent studies of autophagy, which occurs in salivary glands under stress but has not been investigated extensively and has not yet been associated with cystatins in these organs [46]. Cystatin C can promote autophagy in neurons as a protective mechanism against the accumulation of detritus from free-radical damage caused by metabolic stress, and its ability to inhibit the lysosomal cathepsin B is important in this activity [47]. Exercise constitutes a type of metabolic stress, and autophagy would be expected to increase during the recovery of homeostasis [46]. Mice with conditional knockouts of the autophagy gene Atg5 exhibited disrupted secretory granules in salivary acinar cells under basal as well as isoproterenol-stimulated conditions.

Taken together, these data do not provide an answer, but they do suggest one or more types of protective role for cystatins. If salivary cystatins protect the buccal mucosae against microorganisms, why would they increase during exercise? Often referred to as AMPs (antimicrobial peptides), many of the defense elements found in saliva (including α-amylase, lysozyme, lactoferrin, the cathelicidin LL-37 and α-defensins) increase in concentration or secretion rate immediately following short, intense exercise [12,48,49], as we have found for cystatins. A short burst of cystatin secretion might be relevant to raising innate defenses against infection in the upper respiratory tract. It has been suggested that different AMPs may act synergistically, thereby accounting for their effectiveness at low concentrations [50]. An increase with exercise may act to counterbalance a transient depression of immunity. According to some reports [but see other references in 48], exercise induces a decrease in immunoglobulin A (IgA), the major source of salivary antibodies, usually lasting for a few hours following long training bouts or extended competitive efforts such as a marathon [1, 51]. Typically this is interpreted as a sign of suppressed immunity that provides an “open window” of opportunity for upper-respiratory-tract infections [1,29,52,53,54]. A problem with this hypothesis is the difficulty encountered in the literature about finding conditions for reproducing the IgA response, which suggests that there may be factors controlling IgA concentration that are not yet understood [48,53,55]. Certainly it is not clear whether the transient upregulation of defense peptides is sufficient to counter the more prolonged loss of antibody production from IgA [49]. There appears to be a need for more data on this point.

Salivary secretion is highly variable and each gland produces a different volume, as well as a different pattern of proteins [56]. The incorporation (or not) of different proteins into secretory granules provides an additional variable [4]. Thus there may be more than one reason for the differences observed in the concentration changes for α-amylase, cystatins and total protein with relation to the time after the end of an exercise. At our earliest time point (5 min post-exercise), all three components were at their maximal value, but from this point on, their secretory profiles diverged. By 10 min post-exercise, the total protein remained high while the α-amylase had already fallen by ~30% (Fig 6), consistent with a recovery half-time of 10–15 min [39]. In contrast, there was a sharper reduction in secretion of S-type cystatins, which at 10 min was below the resting value (Fig 6). The overall protein concentration remained elevated in saliva through 15 min. One way to account for this pattern would be to imagine small, medium and large pools for cystatins, amylase and total protein, respectively. Other scenarios are also possible, such as different storage packets, different rates of re-synthesis, and different rates of transport to the cell membrane where exocytosis takes place.

When the saliva from physical tests was analyzed for each athlete at the same time point, a large variance in the response of S-type cystatins and cystatin C was observed, ranging from as much as a 9.8-fold increase for one individual to a decrease below resting value for three others (Fig 3). This heterogeneity of expression among individual saliva samples as well as for particular proteins has been found in other studies [48], even at rest [56]. it may reflect a difference in the flux of protein transport or glandular expression capacity between individuals, and when the performance goal is $\dot{V}O_{2\max}$ there could also be an effect due to differences in the training state of the athletes [57]. However, no one athlete maintained consistently low values or consistently high values across all the tests. The cycloergometer data with incremental loads in Fig 4 indicate that near-maximal effort may be a prerequisite for increased levels of cystatin, so a low value might arise for an athlete who was less motivated toward maximal effort on a particular day. Following exercise, accurate timing of saliva collection is another factor in variability: while concerted efforts were made to perform collections within 5 min of the end of an exercise, the rapid return of S-type cystatins to basal levels might also lead to an individual showing no increase due to issues with collection rather than response. Tests with different saliva collection methods did not resolve these questions: passive drool was slow and awkward for the athletes [14], and produced variable amounts of mucus, while spitting produced higher concentrations of total protein, but no improvement in the coefficient of variation (CV). Among 8 sets of data for which volume, cystatin and total protein were measured, the average CV for the volume of saliva (among 12 subjects in each set) was 14%, much smaller than the variability in cystatin signals seen in Fig 3. Collecting unstimulated saliva as described in our protocol would tend to minimize variability due to interactions between oral stimuli, pH and flow rates [15,16,40].

A limitation of our study design is the absence of data on the increase in catecholamines that accompanied our chosen exercises. However, salivary amylase and plasma lactate have both been demonstrated repeatedly to respond during exercise in parallel with the release of catecholamines [30,31,39]. While salivary glands are innervated by parasympathetic as well as sympathetic nerves, the sympathetic signals are related to the types, duration and intensity of exercise as well as timing of sample collection [58]. Here, it was demonstrated that secretion of S-type cystatins and cystatin C increased with exercise lasting between 90 s and 15 min, provided that the physical effort was close to maximal. Our results showing high levels of salivary amylase and plasma lactate are consistent with involvement of the autonomic nervous system in the acute expression and secretion of type-2 cystatins from salivary glands. However, it is still possible that an increase in type-2 cystatin expression or secretion in saliva receives contributions from other tissues, since exercise leads to an increase in sympathoadrenal activity throughout the body [59]. Overall, in the 24 samples collected from the athletes for the two protocols in Fig 3, 83% showed an increase in cystatin secretion in saliva immediately after both aerobic and anaerobic tests. Other studies have recorded changes in cortisol, indicating participation of the hypothalamic-pituitary-adrenal axis, but we have not pursued this aspect [29].

Supporting evidence for cystatin secretion as a response to β-adrenergic stimulation was obtained *in vitro* with HSG, a cell line originally derived from human submandibular glands. Initial experiments with non-differentiated cells showed that the cellular release of cystatin C was stimulated 11 fold by a beta-agonist treatment with IPR. Conversely, the antagonist propranolol decreased production by 9-fold. When cells were plated on Matrigel and allowed to differentiate into acinus-like structures, changes in the subcellular distribution of cystatins type S and cystatin C were tracked by calculating the ratio of peripheral to internal cystatins. Even in unstimulated cell groups, the periphery had a higher fluorescence labeling of cystatin C and cystatins S, suggesting the presence of cystatin-containing vesicles. Activating the β-adrenergic signalling pathway led to a much higher ratio of cystatins at the periphery. It could not be determined whether the IPR treatment accelerated the normal secretion pathway, acted on a separate pool of vesicles or reflected *de novo* production through increased transcription and translation. In rats, IPR increases the exocytosis of α-amylase through a significant increase in interaction between the accessory proteins v- and t-SNARE, which mediate vesicle fusion [60]. It is also possible that catecholamines enhance secretion by accelerating translation of mRNA, as observed in the parotid [61]. More in-depth studies will be required to distinguish among these possibilities.

## Conclusion

Increased concentrations of both cystatin C and cystatins type S were measured in saliva as a response to both aerobic and anaerobic exercise, in association with athletes´ exerting near-maximal effort. The increases in cystatin were rapid and transient, returning to basal levels within 15 min. While increased salivary amylase as well as plasma lactate were consistent with activation of the sympathetic nervous system by the physical activity, the time course of recovery was much faster for cystatin than for amylase or total protein, suggesting different post-secretory processes. An *in-vitro* model showed that the release phase for both cystatins was associated with redistribution of internal stores toward the periphery of acinus-like structures derived from human submandibular glands.

## References

[1] Papacosta E, Nassis GP. Saliva as a tool for monitoring steroid, peptide and immune markers in sport and exercise science. J Sci Med Sport. 2011;14: 424–434. 10.1016/j.jsams.2011.03.004 21474377

[2] de Sousa-Pereira P, Amado F, Abrantes J, Ferreira R, Esteves PJ, Vitorino R. An evolutionary perspective of mammal salivary peptide families: cystatins, histatins, statherin and PRPs. Arch Oral Biol. 2013;58(5): 45145–45148. 10.1016/j.archoralbio.2012.12.011 23352445

[3] Chicharro JL, Lucía A, Pérez M, Vaquero AF, Ureña R. Saliva composition and exercise. Sports Med. 1998;26(1): 17–27. 10.2165/00007256-199826010-00002 9739538

[4] Ekstrom J, Khosravani N, Castagnola M, Messana I. Saliva and control of its secretion In: Olle Ekberg editor. Dysphagia: diagnosis and treatment. Berlin: Springer; 2012 pp. 19–47.

[5] Proctor GB, Carpenter GH. Salivary secretion: mechanism and neural regulation. Monogr Oral Sci. 2014;24: 14–29. 10.1159/000358781 24862591

[6] Henskens YM, Veerman EC, Mantel MS, van der Velden U, Nieuw Amerongen AV. Cystatins S and C in human whole saliva and in glandular salivas in periodontal health and disease. J Dent Res. 1994,73(10): 1606–14. 10.1177/00220345940730100501 7929975

[7] Babad H, Ben-Zvi R, Bdolah A, Schramm M. The mechanism of enzyme secretion by the slices. Eur J Biochem. 1967, 1(1): 96–101. 10.1111/j.1432-1033.1967.tb00049.x 6059351

[8] Kjaer M, Secher NH, Galbo H. Physical stress and catecholamine release. Baillieres Clin Oral Dis. 2018 11;24(8):1477–1483. 10.1111/odi.12920 Epub 2018 Jul 10. 3327495

[9] Bocanegra OL, Diaz MM, Teixeira RR, Soares SS, Espindola FS. Determination of the lactate threshold by means of salivary biomarkers: chromogranin A as novel marker of exercise intensity. Eur J Appl Physiol. 2012;112(9): 3195–3203. 10.1007/s00421-011-2294-4 22227853

[10] Ventre G, Colonna C, Smith J, Alfano D, Moldow R. Salivary VIP concentrations are elevated in humans after acute stress. Peptides. 2013;49: 27–31. 10.1016/j.peptides.2013.08.014 23994551

[11] Aydin S, Aydin S, Kuloglu T, Yilmaz M, Kalayci M, Sahin I, et al Alterations of irisin concentrations in saliva and serum of obese and normal-weight subjects, before and after 45 min of a Turkish bath or running. Peptides. 2013;50: 13–18. 10.1016/j.peptides.2013.09.011 24096106

[12] Ligtenberg AJ, Brand HS, van den Keijbus PA, Veerman EC. The effect of physical exercise on salivary secretion of MUC5B, amylase and lysozyme. Arch Oral Biol. 2015;60(11): 1639–1644. 10.1016/j.archoralbio.2015.07.012 26351746

[13] Henskens YM, van den Keijbus PA, Veerman EC, Van der Weijden GA, Timmerman MF, Snoek CM, et al Protein composition of whole and parotid saliva in healthy and periodontitis subjects. Determination of cystatins, albumin, amylase and IgA. J Periodontal Res. 1996;31(1): 57–65. 10.1111/j.1600-0765.1996.tb00464.x 8636877

[14] CISM—Conseil International du Sport Militaire. Naval Pentathlon: Sports Regulation. Available from: http://www.cism-milsport.org/eng/003_SPORTS/014_naval_pent/NavalPent.pdf, 2009 (accessed 01.10.2015).

[15] Ricardo DR, de Almeida MB, Franklin BA, Araújo CG. Initial and final exercise heart rate transients: influence of gender, aerobic fitness, and clinical status. Chest. 2005;127(1): 318–27. 10.1378/chest.127.1.318 15653999

[16] Sant’Anna ML, Casimiro-Lopes G, Boaventura G, Marques STF, Sorenson MS, Salerno VP.Anaerobic exercise affects the saliva antioxidant/oxidant balance in high-performance pentathlon athetes. Humo. 2016;17(1): 50–55. 10.1515/humo-2016-0003

[17] Zagatto AM, Beck WR, Gobatto CA. Validity of the running anaerobic sprint test for assessing anaerobic power and predicting short-distance performances. J Strength Cond Res. 2009;23(6):1820–7. 10.1519/JSC.0b013e3181b3df32 19675478

[18] Ljungberg G, Ericson T, Ekblom B, Birkhed D. Saliva and marathon running. Scand J Med Sci Sports. 1997;7: 214–219. 9241026 10.1111/j.1600-0838.1997.tb00142.x

[19] Dawes C. The effects of exercise on protein and electrolyte secretion in parotid saliva. J Physiol. 10.1113/jphysiol.1981.sp013940 7320933 PMC1244038

[20] Bellagambi FG, Degano I, Ghimenti S, Lomonaco T, Dini V, Romanelli M, et al Determination of salivary α-amylase and cortisol in psoriatic subjects undergoing the Trier Social Stress Test. Microchem J. 2018 1 136: 177–184. doi.org/10.1016/j.microc.2017.04.033

[21] Navazesh M. Methods for collecting saliva. Ann N Y Acad Sci. 1993;694: 72–7. 10.1111/j.1749-6632.1993.tb18343.x 8215087

[22] Gonçalves Lda R, Soares MR, Nogueira FC, Garcia C, Camisasca DR, Domont G. et al Comparative proteomic analysis of whole saliva from chronic periodontitis patients. Proteomics. 2010;73(7): 1334–1341. 10.1016/j.jprot.2010.02.018 20215060

[23] Bradford MM. Rapid and sensitive method for the quantitation of microgram quantities of protein utilizing the principle of protein-dye binding. Anal Biochem. 1976;72: 248–254. 10.1006/abio.1976.9999 942051

[24] Aguilar HN, Zielnik B, Tracey CN, Mitchell BF. Quantification of rapid Myosin regulatory light chain phosphorylation using high-throughput in-cell Western assays: comparison to Western immunoblots. PLoS One 2010;5(4):e9965. doi.org/10.1371/journal.pone.0009965. 10.1371/journal.pone.0009965 20376358 PMC2848601

[25] Shirasuna K, Sato M, Miyazaki T. A neoplastic epithelial duct cell line established from an irradiated human salivary gland. Cancer (Phila.). 1981;48: 745–752. 7248901 10.1002/1097-0142(19810801)48:3<745::aid-cncr2820480314>3.0.co;2-7

[26] Capes-Davis A, Theodosopoulos G, Atkin I, Drexler HG, Kohara A, MacLeod RA. Check your cultures! A list of cross-contaminated or misidentified cell lines. Int J Cancer. 2010 7 1;127(1):1–8. 10.1002/ijc.25242 20143388

[27] Lin LC, Elkashty O, Ramamoorthi M, Trinh N, Liu Y, Sunavala-Dossabhoy G. Cross-contamination of the human salivary gland HSG cell line with HeLa cells: A STR analysis study. Oral Dis. 2018;24(8):1477–1483. 10.1111/odi.12920 29923277

[28] Schindelin J, Arganda-Carreras I, Frise E, Kaynig V, Longair M, Pietzsch T, et al Fiji: an open-source platform for biological-image analysis. Nat Methods. 2012;9(7): 676–682. 10.1038/nmeth.2019 22743772 PMC3855844

[29] Kakanis MK, Peake J, Brenu EW, Simmonds M, Gray B, Hooper SL, et al The open window of susceptibility to infection after acute exercise in healthy young male elite athletes. Exerc Immunol Rev. 2010;16: 119–137. 20839496

[30] Mikulski T, Ziemba A, Nazar K. Influence of body carbohydrate store modification on catecholamine and lactate responses to graded exercise in sedentary and physically active subjects. J Physiol Pharmacol. 2008;59(3): 603–616 18953101

[31] Scharhag-Rosenberger F, Carlsohn A, Lundby C, Schüler S, Mayer F, Scharhag J. Can more than one incremental cycling test be performed within one day? Eur J Sport. Sci. 2014;14(5): 459–467. 10.1080/17461391.2013.853208 24168437

[32] Fernandes AL, Lopes-Silva JP, Bertuzzi R, Casarini DE, Arita DY, Bishop DJ. Effect of time of day on performance, hormonal and metabolic response. during a 1000-M cycling time trial. PLoS ONE. 2014;9(10): e109954 10.1371/journal.pone.0109954 25289885 PMC4188634

[33] Soltoff S, Hedden LJ. Isoproterenol and cAMP block ERK phosphorylation and enhance [Ca^2+^]i Increases and oxygen consumption by muscarinic receptor stimulation in rat parotid and submandibular acinar cells. Biol Chem. 2010;285(18): 13337–13348. 10.1074/jbc.M110.112094 20207737 PMC2859492

[34] Carpenter GH. The secretion, components, and properties of saliva. Annu Rev Food Sci Technol. 2013;4: 267–276. 10.1146/annurev-food-030212-182700 23464573

[35] Messenger SW, Falkowski MA, Groblewski GE. Ca^2+^-regulated secretory granule exocytosis in pancreatic and parotid acinar cells. Cell Calc. 2014;55(6): 369–375. 10.1016/j.ceca.2014.03.003 24742357 PMC4058401

[36] Chopra DP, Xue-Hu IC. Secretion of alpha-amylase in human parotid gland epithelial cell culture. J Cell Physiol. 1993;155(2): 223–33. 10.1002/jcp.1041550202 8097745

[37] Vag J, Byrne EM, Hughes DH, Hoffman M, Ambudkar I, Maguire P, et al Morphological and functional differentiation of HSG cells: role of extracellular matrix and trpc 1. J Cell Physiol. 2007;212(2): 416–23. 10.1002/jcp.21035 17348017

[38] Castle D, Castle A. Intracellular transport and secretion of salivary proteins. Crit Rev Oral Biol Med. 1998;9(1): 4–22. 9488245 10.1177/10454411980090010301

[39] Chatterton RT, Jr, Vogelsong KM, Lu YC, Ellman AB, Hudgens GA. Salivary α-amylase as a measure of endogenous adrenergic activity. Clin Physiol. 1996;16: 433–448. 10.1111/j.1475-097x.1996.tb00731.x 8842578

[40] Ghimenti S, Lomonaco T, Onor M, Murgia L, Paolicchi A, Fuoco R, et al Measurement of warfarin in the oral fluid of patients undergoing anticoagulant oral therapy. PLoS One. 2011;6(12): e28182 10.1371/journal.pone.0028182 22164240 PMC3229510

[41] Lindh E, Brännström J, Jones P, Wermeling F, Hässler S, Betterle C, et al Autoimmunity and cystatin SA1 deficiency behind chronic mucocutaneous candidiasis in autoimmune polyendocrine syndrome type 1. J Autoimmun. 2013;42: 1–6. 10.1016/j.jaut.2012.10.001 23122533

[42] Imoto Y, Tokunaga T, Matsumoto Y, Hamada Y, Ono M, Yamada T, et al Cystatin SN upregulation in patients with seasonal allergic rhinitis. PLoS ONE. 2013;8(8): e67057 10.1371/journal.pone.0067057 23950865 PMC3741298

[43] Dickinson DP, Thiesse M, Hicks MJ. Expression of type 2 cystatin genes CST1-CST5 in adult human tissues and the developing submandibular gland. DNA Cell Biol. 2002;21(1): 47–65. 10.1089/10445490252810311 11879580

[44] Ganeshnarayan K, Velliyagounder K, Furgang D, Fine DH. Human salivary cystatin SA exhibits antimicrobial effect against Aggregatibacter actinomycetemcomitans. J Periodont Res 2012; 47: 661–673. 10.1111/j.1600-0765.2012.01481.x 22582873

[45] Blankenvoorde MF, Henskens YM, van't Hof W, Veerman EC, Nieuw Amerongen AV. Inhibition of the growth and cysteine proteinase activity of Porphyromonas gingivalis by human salivary cystatin S and chicken cystatin. Biol Chem. 1996;377(12): 847–850. 8997496

[46] Morgan-Bathke M, Lin HH, Chibly AM, Zhang W, Sun X, Chen CH, et al Deletion of ATG5 shows a role of autophagy in salivary homeostatic control. J Dent Res 2013; 92(10):911–917. 10.1177/0022034513499350 23884556 PMC3775371

[47] Watanabe S, Komine O, Endo F, Wakasugi K, Yamanaka K, et al Intracerebroventricular administration of Cystatin C ameliorates disease in SOD1-linked amyotrophic lateral sclerosis mice. J Neurochem. 2018;145(1): 80–89. 10.1111/jnc.14285 29282717 PMC5947136

[48] West NP, Pyne DB, Kyd JM, Renshaw GM, Fricker PA, Cripps AW. The effect of exercise on innate mucosal immunity. Br J Sports Med. 2010;44: 227–231. 10.1136/bjsm.2008.046532 18499767

[49] Davison G, Allgrove J, Gleeson M. Salivary antimicrobial peptides (LL-37 and alpha-defensins HNP1–3), antimicrobial and IgA responses to prolonged exercise. Eur J Appl Physiol. 2009;106: 277–284. 10.1007/s00421-009-1020-y 19263072

[50] Fábián TK, Hermann P, Beck A, Fejérdy P, Fábián G. Salivary defense proteins: their network and role in innate and acquired oral immunity. Int J Mol Sci. 2012;13(4): 4295–4320. 10.3390/ijms13044295 22605979 PMC3344215

[51] Nieman DC. Exercise, Infection, and Immunity Int. J. Sports Med. 1994(15): S131—S141. 7883395 10.1055/s-2007-1021128

[52] Nieman DC. Risk of upper respiratory tract infection in athletes: an epidemiologic and immunologic perspective. J Athl Train. 1997;32(4): 344–349. 16558471 PMC1320353

[53] Walsh NP, Gleeson M, Shephard RJ, Gleeson M, Woods JA, Bishop NC, et al Position statement. Part one: Immune function and exercise. Exerc Immunol Rev. 2011;17: 6–63 21446352

[54] Gleeson M, David BP, Lisa JE, Sharron TH, John RA, Christopher O, et al Developing a multi-component immune model for evaluating the risk of respiratory illness in athletes Exerc Immunol Rev. 2017;23:52–64. 28230530

[55] Campbell JP, Turner JE. Debunking the myth of exercise- induced immune suppression: redefining the impact of exercise on immunological health across the lifespan. Front. Immunol. 2018;9: 648 10.3389/fimmu.2018.00648 29713319 PMC5911985

[56] Jasim H, Olausson P, Hedenberg-Magnusson B, Ernberg M, Ghafouri B. The proteomic profile of whole and glandular saliva in healthy pain-free subjects Scientific Reports. 2016;6: 39073 10.1038/srep39073 27976689 PMC5157045

[57] Kunz H, Bishop NC, Spielmann G,·Pistillo M, Reed J, Ograjsek T, et al ·Fitness level impacts salivary antimicrobial protein responses to a single bout of cycling exercise. Eur J Appl Physiol. 2015;115: 1015–1027. 10.1007/s00421-014-3082-8 25557386

[58] Zouhal H, Jacob C, Delamarche P, Gratas-Delamarche A. Catecholamines and the effects of exercise, training and gender. Sports Med. 2008;38(5): 401–423. 10.2165/00007256-200838050-00004 18416594

[59] Ji X, Yao L, Wang M, Liu X, Peng S, Li K, et al Cystatin C attenuates insulin signaling transduction by promoting endoplasmic reticulum stress in hepatocytes. FEBS Lett. 52015;589(24): 3938–3944. 10.1016/j.febslet.2015.11.029 26592151

[60] Takuma T, Shitara A, Arakawa T, Okayama M, Mizoguchi I, Tajima Y. Isoproterenol stimulates transient SNAP23-VAMP2 interaction in rat parotid glands. FEBS Lett. 2013;587(6): 583–9. 10.1016/j.febslet.2013.01.039 23380067

[61] Kim SK, Jones TP, Cuzzort LM. Protein synthesis and amylase messenger RNA content in rat parotid salivary glands after total or partial stimulation with isoproterenol. Arch Oral Biol. 1989;34(11): 895–901. 10.1016/0003-9969(89)90147-7 2482020

